# MicroRNA-152-3p and MicroRNA-196a-5p Are Downregulated When Müller Cells Are Promoted by Components of the Internal Limiting Membrane: Implications for Macular Hole Healing

**DOI:** 10.3390/ijms242417188

**Published:** 2023-12-06

**Authors:** Hung-Da Chou, Shine-Gwo Shiah, Lan-Hsin Chuang, Wei-Chi Wu, Yih-Shiou Hwang, Kuan-Jen Chen, Eugene Yu-Chuan Kang, Ling Yeung, Chung-Yi Nien, Chi-Chun Lai

**Affiliations:** 1 Department of Life Sciences, National Central University, Taoyuan 32001, Taiwan; hungda.chou28@gmail.com (H.-D.C.); davidssg@nhri.edu.tw (S.-G.S.); 2 Department of Ophthalmology, Chang Gung Memorial Hospital, Linkou Main Branch, Taoyuan 33305, Taiwan; weichi666@gmail.com (W.-C.W.); yihshiou.hwang@gmail.com (Y.-S.H.); cgr999chiayi@yahoo.com.tw (K.-J.C.); yckang0321@gmail.com (E.Y.-C.K.); 3 College of Medicine, Chang Gung University, Taoyuan 33305, Taiwan; lanhsin.chuang@gmail.com (L.-H.C.); lingyeung@gmail.com (L.Y.); 4 National Institute of Cancer Research, National Health Research Institutes, Miaoli 35053, Taiwan; 5 Department of Ophthalmology, Chang Gung Memorial Hospital, Keelung 20401, Taiwan

**Keywords:** microRNA, macular hole, internal limiting membrane, Müller cells, miR-152-3p, miR-196a-5p

## Abstract

Müller cells play a critical role in the closure of macular holes, and their proliferation and migration are facilitated by the internal limiting membrane (ILM). Despite the importance of this process, the underlying molecular mechanism remains underexplored. This study investigated the effects of ILM components on the microRNA (miRNA) profile of Müller cells. Rat Müller cells (rMC-1) were cultured with a culture insert and varying concentrations of ILM component coatings, namely, collagen IV, laminin, and fibronectin, and cell migration was assessed by measuring cell-free areas in successive photographs following insert removal. MiRNAs were then extracted from these cells and analyzed. Mimics and inhibitors of miRNA candidates were transfected into Müller cells, and a cell migration assay and additional cell viability assays were performed. The results revealed that the ILM components promoted Müller cell migration (*p* < 0.01). Among the miRNA candidates, miR-194-3p was upregulated, whereas miR-125b-1-3p, miR-132-3p, miR-146b-5p, miR-152-3p, miR-196a-5p, miR-542-5p, miR-871-3p, miR-1839-5p, and miR-3573-3p were significantly downregulated (*p* < 0.05; fold change > 1.5). Moreover, miR-152-3p and miR-196a-5p reduced cell migration (*p* < 0.05) and proliferation (*p* < 0.001), and their suppressive effects were reversed by their respective inhibitors. In conclusion, miRNAs were regulated in ILM component-activated Müller cells, with miR-152-3p and miR-196a-5p regulating Müller cell migration and proliferation. These results serve as a basis for understanding the molecular healing process of macular holes and identifying potential new target genes in future research.

## 1. Introduction

Idiopathic full-thickness macular hole (FTMH) is a vision-threatening condition among older individuals [1,2,3]. Despite its relatively small average diameter of 400 µm, the fact that its critical location is at the fovea, which is responsible for central vision, renders FTMH capable of inducing permanent visual impairment if left unattended [3]. Although the precise pathophysiology of FTMH remains incompletely understood, it is believed to involve vitreous degeneration and abnormal traction in the vitreomacular interface [4]. Studies have suggested that the Müller cells in the fovea are key contributors to the formation of FTMH [4,5]. Müller cells, the primary glial cell type in the retina, play a crucial role in providing structural and metabolic support to the neurosensory retina [6,7]. During the initial stages of macular hole development, the “foveola Müller cell cone” experiences traction and detaches from the parafoveal Müller cell wall. A full-thickness hole is formed when the foveola Müller cell cone is fragmented, resulting in a break in the outer retinal layers [5].

Most FTMHs necessitate surgical intervention, typically through vitrectomy, to alleviate vitreous tractions. The primary closure rate after vitrectomy was reported to be 79–95% for large macular holes. The success rate of this procedure can be further enhanced by harvesting an internal limiting membrane (ILM) flap to cover the hole [8,9,10]. The ILM is believed to act as a scaffold, facilitating the proliferation and migration of Müller cells and promoting Müller cell activation to enhance MH closure [11]. The main components of the ILM include collagen IV, laminin, and fibronectin [12]. In the in vitro model, the ILM components have been indicated to enhance the survival, proliferation, and migration of mammalian Müller cells [11,13], which are critical in the healing process of the macular hole [14,15]. Consequently, our interest lies in further characterizing Müller cells, particularly when influenced by the components of the ILM.

MicroRNAs (miRNAs) constitute a class of small, noncoding RNAs of approximately 22 nucleotides in length. These miRNAs target mRNA by binding to its 3′untranslated region (3′UTR) through miRNA seed sequences (typically 6–8 nucleotides long). Most miRNAs play roles in posttranslational gene regulation through translational repression or mRNA degradation [16]. Since their discovery in the early 1990s, miRNAs have emerged as key players involved in a wide array of biological processes. miRNA dysregulation has been observed in various diseases, including cancer, cardiovascular diseases, diabetes, and neurodegenerative diseases [17,18,19,20,21]. The highly conserved nature and small size of miRNAs have made them potential targets for investigation and therapeutic interventions [22,23].

Ocular studies have reported the involvement of miRNA in vitreoretinopathies, encompassing conditions such as retinal detachment, macular holes, macular degeneration, and diabetic retinopathy [24,25,26,27]. The structural integrity of the retina was also significantly affected by changes in miRNA [28]. However, to the best of our knowledge, the specific role of miRNA in Müller cell migration and proliferation remains unexplored. Therefore, this study aimed to elucidate the miRNA profile of Müller cells when stimulated by ILM components and to explore the associated cellular and molecular functions underlying these processes.

We identified 10 regulated miRNAs, and through miRNA mimic and inhibitor transfection assays, we demonstrated that both miR-152-3p and miR-196a-5p exert inhibitory effects on Müller cell migration and proliferation. Notably, the suppressive actions of these miRNAs were effectively reversed by their respective inhibitors. MiR-152-3p has been reported to regulate cellular proliferation, invasion, and extracellular matrix expression by targeting forkhead box protein F1 (FOXF1) in keloid fibroblasts [29]. In the eyes, miR-152-3p upregulation was observed in the retina and choroid during the vasoobliteration phase in the oxygen-induced retinopathy model [30]. Furthermore, miR-152-3p was found to be regulated in the vitreous humor of eyes with neovascular age-related macular degeneration [31]. As for miR-196a-5p, it has been implicated in targeting forkhead box protein O1 (FOXO1) and has been associated with various cancers [32,33,34,35]. In lens epithelial cells, miR-196a-5p upregulation reduced the level of oxidative stress-induced apoptosis [36]. By identifying these miRNAs, we provide supplementary information on the regulation of Müller cells and provide a foundation for further research in this context.

## 2. Results

### 2.1. Effect of ILM Components in Müller Cell Migration

**Figure 1:**
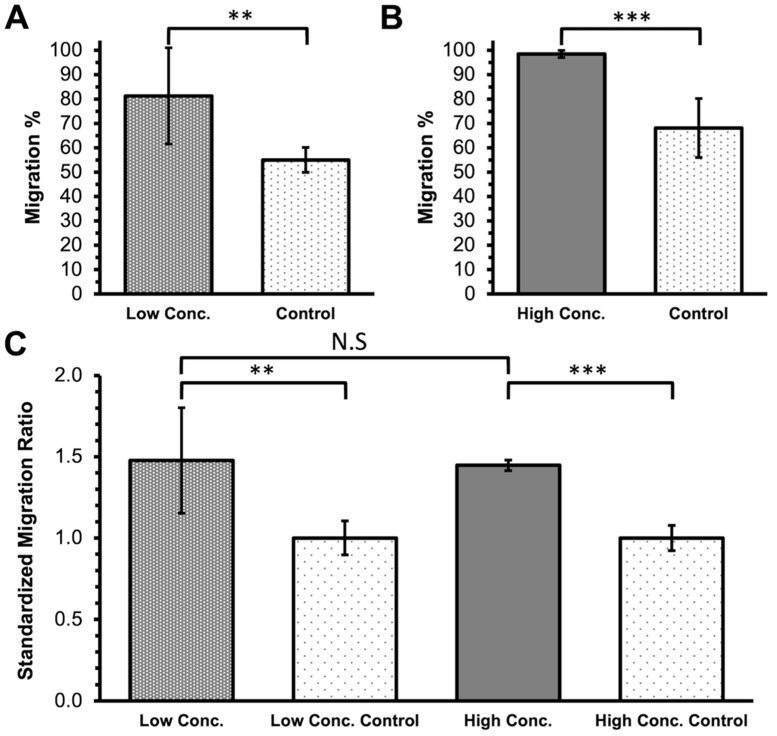
Migration of rat Müller cells cultured on low and high concentrations (conc.) of internal limiting membrane component coatings, including collagen IV, laminin, and fibronectin. (**A**,**B**) Müller cells exhibited a significant increase in migration in both the low conc. group and the high conc. group compared with the controls. (**C**) The standardized comparison between the low and high conc. groups revealed no significant difference (*p* = 0.414). (** *p* < 0.01; *** *p* < 0.001; N.S., nonsignificant; and *N* = 6 in each group.).

To assess the effect of varying concentrations of ILM components on the stimulation of the Müller cells, a lower concentration (collagen IV, 10 µg/cm^2^; laminin, 20 µg/mL; fibronectin, 20 µg/mL) and a higher concentration (collagen IV, 20 µg/cm^2^; laminin, 30 µg/mL; fibronectin, 100 µg/mL) of the ILM component were used to coat the culture plate before Müller cell seeding. Both concentration groups and the control group were subject to the migration assay. In the low-concentration group, Müller cells exhibited significantly increased migration compared with the control group (*p* < 0.01; Figure 1A). Similarly, in the high-concentration group (Figure 1B), significantly greater migration activities were observed compared with the control group (*p* < 0.001). However, after standardization with respect to the control (Figure 1C), no significant difference was noted between the low- and high-concentration groups (1.48 ± 0.32 vs. 1.45 ± 0.03; *p* = 0.414). These findings suggest that the combination of collagen IV, laminin, and fibronectin promotes Müller cell migration. However, no significant dose-dependent effect was observed between the two concentrations used in this study.

### 2.2. MiRNA Profiling of Müller Cell Stimulated by ILM Components

**Figure 2:**
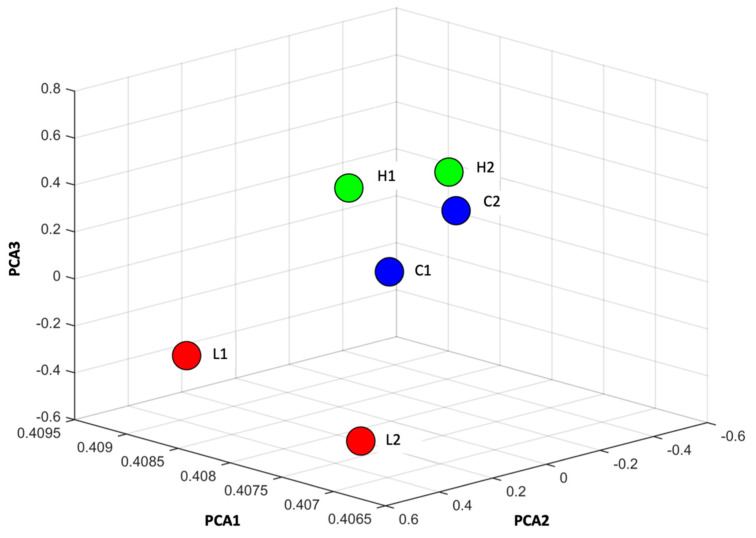
Principal component analysis (PCA) plot of the microRNA profiles of rat Müller cells cultured on low- and high-concentration internal limiting membrane component coatings. The controls (C1 and C2, blue) and the low-concentration group (L1 and L2, red) exhibit distinct clustering. The high-concentration group (H1 and H2, green) also forms clusters, with some overlap observed with the controls, particularly between H2 and C2.

**Figure 3:**
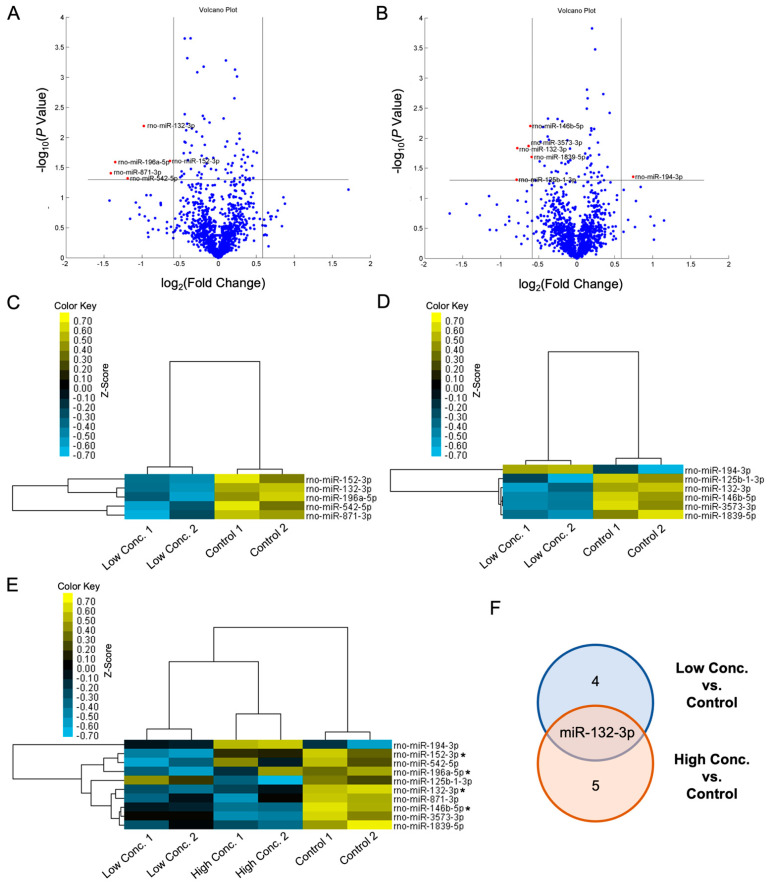
Differentially expressed microRNAs (miRs) in rat Müller cells harvested from different internal limiting membrane component concentration (conc.) groups. (**A**,**B**) Volcano plots depicting log fold changes plotted against *p* values in samples from the low conc. (**A**) and high conc. (**B**) groups versus the control. Red dots indicate significantly regulated miRs (*p* < 0.05 and log fold change > 1.5). (**C**–**E**) Hierarchical clustering heatmaps illustrate the differentially expressed miRs. Each row and column indicate a miR and one sample, respectively. The bars indicate relative expression levels from high (yellow) to low (blue). The asterisks in (**E**) indicate the miRs selected for the further transfection assays. (**F**) The Venn diagram indicates that miR-132-3p is downregulated in both the low and high conc. groups.

Following stimulation, Müller cells were harvested and subjected to miRNA profiling. In total, 1218 miRNAs were identified. A three-dimensional principal component analysis (PCA) plot based on miRNA expression (Figure 2) revealed distinct clustering of the controls, separated from the low-concentration groups. Interestingly, the high-concentration groups were clustered close to the controls. These results suggest that the miRNA profile of the lower-concentration group differed more significantly from the controls, whereas the high-concentration group was less distinguishable from the controls. Volcano plots were generated to illustrate the differentially expressed miRNAs from the miRNA microarray analysis (Figure 3). With a threshold of *p* < 0.05 and a fold change of at least 1.5, five and six miRNAs were identified from the low- and high-concentration groups, respectively. In the low-concentration group, five miRNAs, namely miR-132-3p, miR-152-3p, miR-196a-5p, miR-542-5p, and miR-871-3p, were significantly downregulated (Figure 3A), and in the high-concentration group, five miRNAs (miR-125b-1-3p, miR-132-3p, miR-146b-5p, miR-1839-5p, and miR-3573-3p) were downregulated and one (miR-194-3p) was upregulated (Figure 3B). In general, the fold change of the significantly regulated miRNAs in the high-concentration group was lower than that in the low-concentration group.

Clustered heatmaps were generated to visualize the expression patterns of differentially expressed miRNAs modulated with ILM components. In both the low- and high-concentration groups, the replicates of each condition exhibited well-defined clustering (Figure 3C,D). However, in the combined heatmap, variations were observed in miR-542-5p and miR-196a-5p between the two replicates within the high-concentration group (Figure 3E). Furthermore, different miRNA regulation was evident between the low- and high-concentration groups. For example, miR-194-3p and miR-152-3p were downregulated in the low-concentration group but upregulated in the high-concentration group. Conversely, miR-125b-1-3p demonstrated opposite regulation between the two groups (Figure 3E).

Finally, the Venn diagram illustrated that the only overlapping miRNA between the low- and high-concentration groups was miR-132-3p (Figure 3F). In addition to miR-132-3p, three additional miRNAs (miR-146b-5p, miR-152-3p, and miR-196a-5p) were selected for further transfection assays based on the results of the clustering heatmaps. These miRNAs exhibited more consistent expression in both the low- and high-concentration groups, and additional information on them is available in the literature.

### 2.3. Transfection of MiRNA Mimics and Inhibitors

**Figure 4:**
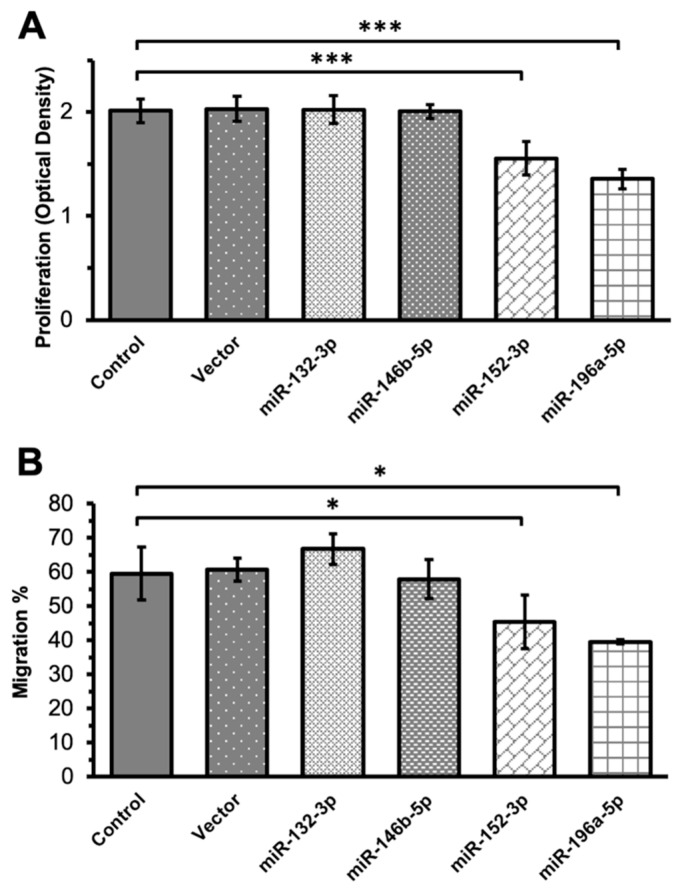
Transfection assays based on four selected microRNA (miRs) mimics. Rat Müller cells cultured with miR-152-3p or miR-196a-5p mimics exhibit a significant suppression of cell proliferation (**A**) and migration (**B**). (* *p* < 0.05; *** *p* < 0.001; and *N* = 6 in each group).

**Figure 5:**
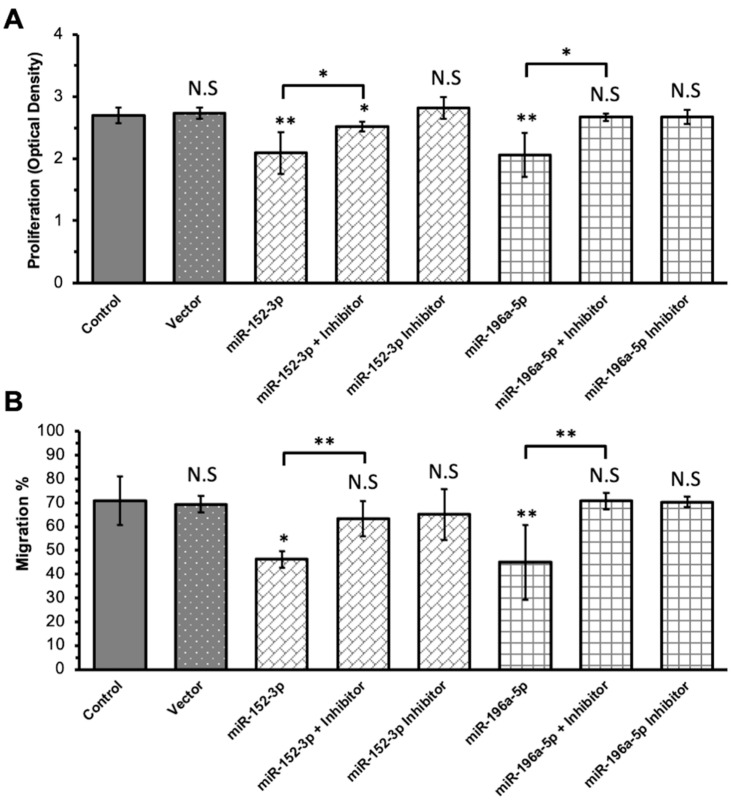
Effect of microRNA (miR) inhibition on the proliferation and migration of rat Müller cells. (**A**) The suppression of Müller cell proliferation via miR-152-3p and miR-196a-5p mimics is partially reversed with the addition of a miR-152-3p inhibitor and completely reversed with the addition of a miR-196a-5p inhibitor. (**B**) The suppression of Müller cell migration via miR-152-3p and miR-196a-5p mimics is reversed with the addition of respective inhibitors. (* *p* < 0.05; ** *p* < 0.01; and N.S. = nonsignificant. The *p* values were calculated by comparing them to the control group, unless labeled using quotation marks. *N* = 6 and 4 in each group of the proliferation and migration assays, respectively).

In the transfection assays, ILM components were not used to stimulate the Müller cells. The transfection of Müller cells with miR-132-3p and miR-146b-5p mimics yielded comparable results to the controls regarding proliferation and migration. However, transfection with miR-152-3p and miR-196a-5p mimics resulted in significantly decreased proliferation and migration, indicating a potential regulatory role for these miRNAs in Müller cells (Figure 4). To further elucidate the role of miR-152-3p and miR-196a-5p in Müller cell migration and proliferation, corresponding miRNA inhibitors were constructed. Subsequent migration assays and additional proliferation assays were conducted (Figure 5). The proliferation assays revealed that miR-152-3p and miR-196a-5p mimics reduced Müller cell proliferation, with the effect partially reversed by the addition of a miR-152-3p inhibitor and completely reversed by the addition of a miR-196a-5p inhibitor, respectively (Figure 5A). The migration assays revealed similar results: the migration of Müller cells was downregulated via miR-152-3p and miR-196a-5p mimics and counteracted with miR-152-3p and miR-196a-5p inhibitors, respectively (Figure 5B). These findings indicate that both miR-152-3p and miR-196a-5p played regulatory roles in the migration and proliferation of Müller cells.

### 2.4. Prediction of Target Genes for Selected MiRNAs

**Figure 6:**
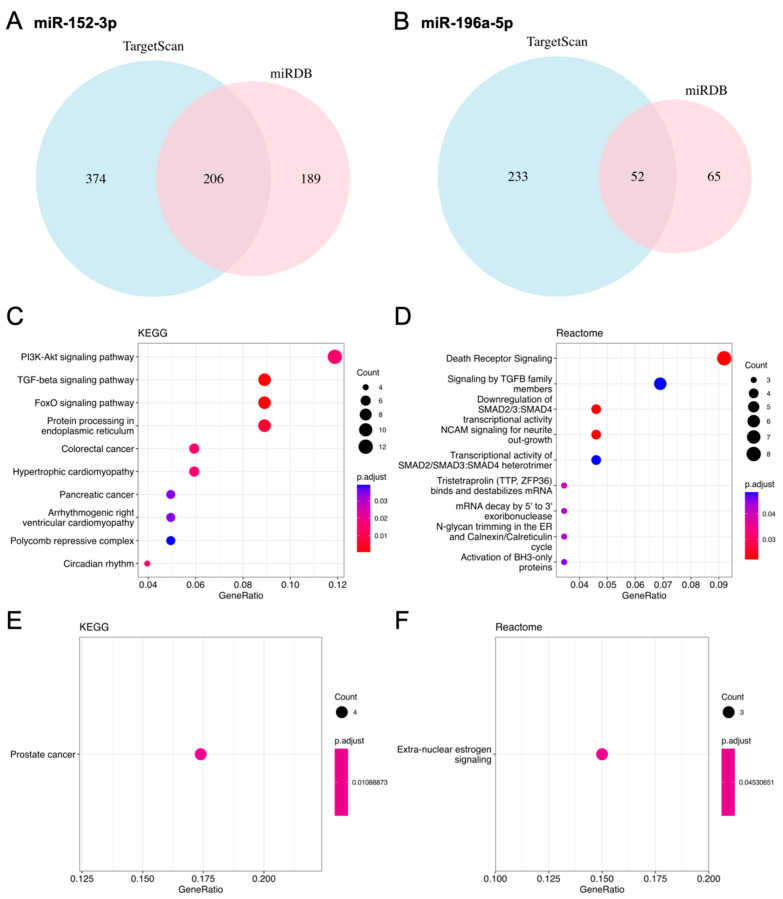
Predicted targets of microRNA (miR)-152-3p and miR-196a-5p and results of function and pathway analyses. (**A**) MiR-152-3p targets. (**B**) MiR-196a-5p targets. Blue depicts the predicted gene number from TargetScan, and pink denotes the number from miRDB. (**C**,**D**) The Kyoto Encyclopedia of Genes and Genomes (KEGG) and Reactome-enriched pathways of miR-152-3p targets. (**E**,**F**) The KEGG and the Reactome-enriched pathways of miR-196a-5p targets.

The potential targets of selected miR-152-3p and miR-196a-5p were predicted using TargetScan and miRDB. The overlapping genes from the two algorithms resulted in 206 and 52 predicted targets for miR-152-3p and miR-196a-5p, respectively (Figure 6A,B). Subsequent Kyoto Encyclopedia of Genes and Genomes (KEGG) and Reactome analysis revealed that the predicted target genes of miR-152-3p were associated with pathways including death receptors, phosphatidylinositol 3-kinase/protein kinase B (PI3K/AKT), transforming growth factor-β (TGF-β), and forkhead box protein O (FOXO) signaling (Figure 6C,D). Conversely, the associated pathways for miR-196a-5p were more limited (Figure 6E,F).

## 3. Discussion

The current study demonstrated that the ILM components effectively promoted the migration of Müller cells. However, no significant differences were observed between the groups with high and low concentrations of the tested ILM components. The miRNA profile of Müller cells promoted by ILM components differed from that of the control group, with one upregulated and ten downregulated miRNAs. Subsequent transfection assays of synthetic miRNA mimics revealed that miR-152-3p and miR-196a-5p suppressed the proliferation and migration of Müller cells. This inhibitory effect was successfully reversed by the respective miRNA inhibitors, confirming the suppressive role of miR-152-3p and miR-196a-5p.

The advent of optical coherence tomography has enabled clinicians and researchers to observe the healing of macular holes in vivo. Whether through spontaneous closure or closure following surgical intervention, gliotic tissue, predominantly formed by Müller cells, has been identified to bridge the hole edges and regress when the outer retinal layers heal [4,15]. However, in some situations, the gliotic tissue occupies the hole and forms scar tissue, thus preventing the coalescence of the photoreceptor layer and interfering with the recovery of vision [4,37].

The regulation of Müller cells and associated gliosis has garnered attention, given that Müller cells are the primary responders to almost all retinal injuries and degenerations and have the potential for neuron regeneration [38,39,40]. In response to laser-induced retinal injuries, Müller cells instantly become reactive and exhibit increased expression of glial fibrillary acidic protein (GFAP), a key component contributing to the mechanical stiffness of the glial cell processes. Additionally, reactive Müller cells are capable of re-entering the cell cycle in preparation for proliferation [41]. In response to retinal injuries, Müller cells undergo three primary gliotic changes—hypertrophy, proliferation, and migration—to protect the remaining healthy tissue [42]. A temporal window exists before gliosis transitions into a chronic scar, during which directing Müller cells in gliotic tissue to dedifferentiate into progenitor cells and regenerate retinal neurons, as observed in lower vertebrates, may be plausible. However, to date, such investigations have yet to be translated to clinical applications [43].

To understand the clinical observation indicating that ILM enhances FTMH closure, the interaction between ILM and Müller cells has been investigated by various research groups. We previously demonstrated that ILM components promoted the migration of rat Müller cells in vitro, and this promotion was significantly enhanced when combined with neurotrophic factors including epidermal growth factor, fibroblast growth factor (FGF), and insulin-like growth factor 1 (IGF-1) [13]. Although we did not examine the effect of ILM components on the proliferation of the Müller cells, co-culturing rabbit Müller cells with ILM was found to enhance their proliferation through the PI3K/AKT pathway, and this effect was further strengthened by the nerve growth factor, another neurotrophic factor [44]. Another model using human Müller cells also demonstrated the promotion of Müller cell proliferation and survival by ILM [45]. Moreover, human Müller cells, activated by ILM components, produced more neurotrophic factors compared with nonactivated Müller cells [11]. These findings align with the aforementioned studies, confirming that Müller cells are promoted by ILM components. However, within the two concentrations tested, the higher concentration of ILM components did not further promote the migration of Müller cells compared with the lower-concentration group. This result may partially explain the variation in clinical outcomes of gliosis even with the placement of an ILM flap, suggesting that a precise amount of ILM components may be necessary to induce the desired healing process for FTMHs.

Among the selected ILM-modulated miRNAs, both miR-152-3p and miR-196-5p exhibited suppressive effects on the proliferation and migration induced by ILM. MiR-152-3p is of particular interest due to its regulatory role in cellular apoptosis, proliferation, or migration in both normal and cancerous cells [46,47,48,49]. Predicted target genes were associated with pathways including death receptors, PI3K/AKT, transforming growth factor-β (TGF-β), and FOXO signaling (Figure 6A,B). In the present study, miR-152-3p was downregulated upon Müller cell activation, potentially reducing death receptor signaling and consequently increasing the cellular viability of Müller cells. In line with the predicted pathway of miR-152-3p, studies on Müller cell proliferation have emphasized the necessity of PI3K/AKT for robust Müller glial proliferation [44,50]. Furthermore, another predicted signaling pathway (TGF-β2) was demonstrated to be upregulated by Müller-cell-related glia [51]. MiR-196-5p was also demonstrated to suppress Müller cell migration and proliferation in the current study. However, the Gene Ontology (GO) enrichment analysis revealed limited related pathways (Figure 6C,D), and studies on the association between miR-196-5p and Müller cells are limited. Nevertheless, miR-196-5p has been associated with aberrant regulation of the PI3K/AKT signaling in lung cancer cells and the behaviors of tumor cells, including glioma and colorectal cancer [33,34,35]. Transcriptome profiling of ILM-modulated Müller cells and function analysis would further validate these results and deepen our understanding of reactive Müller cells mediated via ILM.

The study findings suggest that miR-152-3p and miR-196-5p are associated with the regulation of cellular proliferation, migration, or apoptosis. However, the limitations of the current study should be acknowledged. The retina is a complex structure, and Müller cells are just one component of it. Therefore, the results obtained may not be directly applicable to macular holes. Laser-induced retinal injury, although commonly adopted to establish animal models for retinal pathologies, may induce excessive inflammation and impair choroidal perfusion beneath the lesion, which may not accurately mimic idiopathic FTMHs [52,53]. Nonhuman primates, which more closely resemble humans, are more ideal models for FTMH studies and can be considered in future research [11,54]. Additionally, the identified miRNAs were not confirmed through a polymerase chain reaction in the current study. Nevertheless, the use of specific miRNA mimics and inhibitors in Müller cell proliferation and migration assays yielded consistent results, indicating that the identified differentially expressed miRNAs play a role in regulating these cellular processes.

## 4. Materials and Methods

### 4.1. Cell Line

The study was performed in accordance with the ethical standards outlined in the 1964 Declaration of Helsinki. Rat Müller cells (rMC-1; #RRID: CVCL_8140) were obtained from Professor Vijay Sarthy’s laboratory (Northwestern University, Evanston, IL, USA) [55] and were cultured with Dulbecco’s Modified Eagle’s Medium and Ham’s F-12 (DMEM/F12) containing 10% fetal bovine serum (FBS), 1× penicillin–streptomycin, and 2 mM of L-glutamine (Gibco, Grand Island, NY, USA).

### 4.2. Cell Migration Assay

**Figure 7:**
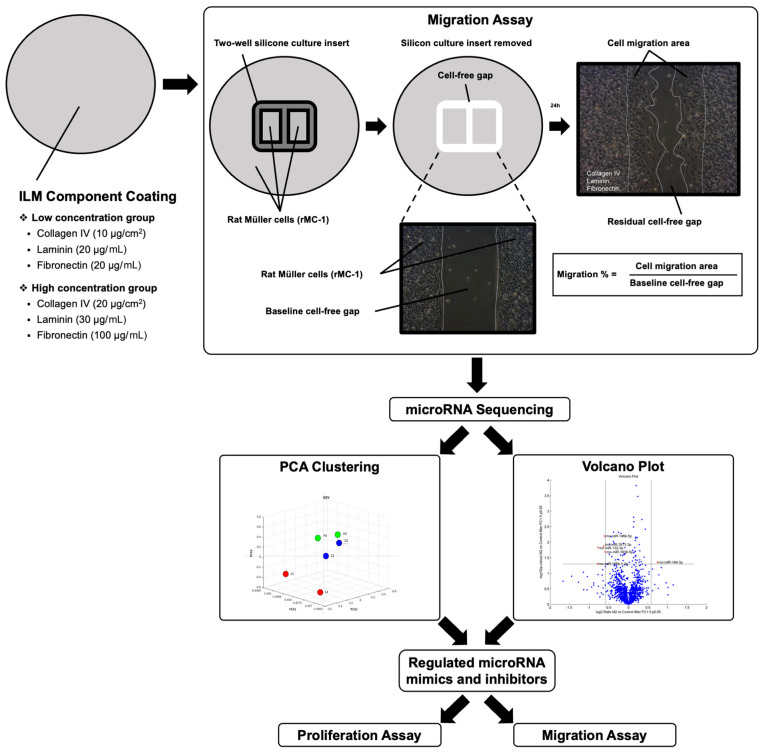
Illustration of the assays conducted in the current study. The cell culture plates were first coated with either low or high concentrations of internal limiting membrane components, including collagen IV, laminin, and fibronectin. Subsequently, migration assays are conducted using Müller cells collected from the migration assays. The Müller cells are then subjected to microRNA sequencing. The obtained microRNA profile is analyzed and clustered through principal component analysis (PCA), and volcano plots are generated. Finally, proliferation and migration assays are conducted using microRNA mimics and/or inhibitors.

To assess the effect of ILM components on Müller cell migration, culture insert migration assays were employed (Figure 7). Six-well culture plates were coated with either low or high concentrations of ILM components. The low-concentration group comprised 10 µg/cm^2^ collagen IV (Advanced BioMatrix, San Diego, CA, USA), 20 µg/mL laminin (Sigma-Aldrich, Saint Louis, MO, USA), and 20 µg/mL fibronectin (Advanced BioMatrix, San Diego, CA, USA), and the high-concentration group comprised 20 µg/cm^2^ collagen IV, 30 µg/mL laminin, and 100 µg/mL fibronectin. A two-well culture insert (ibidi GmbH, Grafelfing, Germany) served as a barrier for cell growth and was placed at the center of each culture plate before seeding Müller cells at a density of 10^6^ cells/mL (70 μL volume). Subsequently, Müller cells were cultured to 100% confluence in DMEM/F12 and 10% FBS under standard laboratory conditions. A minimum of 24 h was allowed for adequate cell attachment before carefully removing the culture insert, creating two square-shaped 500 μm cell-free gaps. Baseline images of the cell-free gaps were captured using a microscope (TS100, Nikon, Tokyo, Japan) equipped with a digital camera (DS-Fi1, Nikon, Tokyo, Japan), and these images were marked with a colored pen at the back of the plates. After 24 h, with the use of the same settings, the migration of Müller cells into the cell-free area was imaged. An imaging software (PhotoImpact v8.0, Ulead Systems, Taipei, Taiwan) was used to evaluate the cell-covered area in the photographs, and the percentage of migration was calculated by dividing the cell migration area at 24 h by the baseline cell-free gap area. Six replicates were performed in each group.

### 4.3. RNA Purification and MiRNA Profiling

To investigate the regulatory changes in Müller cells induced by ILM components, miRNA profiling was performed on the cultured Müller cells obtained from the aforementioned migration assay. Total RNA was extracted and purified using the TRIzolTM Plus RNA Purification Kit (Thermo Fisher Scientific, Waltham, MA, USA) in accordance with the manufacturer’s instructions. Subsequently, the miRNA landscape was probed using miRNA microarrays (Applied Biosystems GeneChip miRNA 4.0 Array, ThermoFisher Scientific, Waltham, MA, USA; #902412) for the control group (*n* = 2), low-concentration group (*n* = 2), and high-concentration group (*n* = 2) per the standard manufacturer’s protocol. The raw microarray datasets were analyzed using the Applied Biosystems Transcriptome Analysis Console 4.0, and miRNA expression levels were summarized using the “RMA+DABG (Rat Only)” algorithm. The resulting datasets were employed for PCA and clustering. The differentially expressed miRNAs between the control and the low/high-concentration groups were identified based on a *p* value of <0.05 and an absolute fold change of >1.5.

### 4.4. Transfection of MiRNA Mimics and Inhibitors Followed by Migration Assay

To proceed with transfection, several differentially expressed miRNAs were selected, and synthetic mimics and inhibitors were used (Table 1). First, 3.0 μL (10 µM) of miRNA mimics/inhibitors (Biotools, Taipei, Taiwan) and 9.0 μL of transfection reagent (Lipofectamine RNAiMAX Reagent, ThermoFisher Scientific, Waltham, MA, USA) were individually diluted in 150 μL of F12-serum-free medium. Subsequently, the two solutions were combined and incubated at room temperature for 5 min, resulting in the formation of 300 μL of miRNA mimics/inhibitors–lipid complex.

Next, the cell migration assay was performed per the aforementioned procedure, but with the following modifications: Müller cells were cultured to 60–80% confluence in DMEM/F12 and 10% FBS in 6-well plates without the ILM component coatings. Subsequently, 250 µL of the miRNA mimics/inhibitors–lipid solution was added per well in the experimental group. In the vector control group, the diluted transfection reagent was added alone to the culture plate without miRNA mimics/inhibitors. Six replicates were performed in each group. Following transfection, an additional 1–3 days of incubation were allowed to reach 100% confluence before conducting the cell migration assay.

### 4.5. Cell Proliferation Assay

To further assess the effect of miRNA mimics and/or inhibitors on Müller cell proliferation, a 3-(4,5-dimethylthiazol-2-yl)-2,5-diphenyl-2H-tetrazolium bromide (MTT) assay with water-soluble tetrazolium-1 (WST-1) was used. Ninety-six-well plates were used and filled with culture medium (DMEM/F12 and 10% FBS) along with either 10 µg/cm^2^ collagen IV + 20 µg/mL laminin + 20 µg/mL fibronectin (low-concentration group) or 20 µg/cm^2^ collagen IV + 30 µg/mL laminin + 100 µg/mL fibronectin (high-concentration group) or culture medium only (control group). Müller cells at a density of 1 × 10^4^ per plate were seeded and allowed to proliferate at 37 °C for 24 h. Subsequently, the cells were rinsed three times using phosphate-buffered saline, and excess solution was removed. Next, 10 μL of WST-1 and 100 μL of culture medium (10% FBS and DMEM/F12) per well were added. The cells were then incubated in a humidified incubator for 60 min. During this incubation period, WST-1 was cleaved with mitochondrial dehydrogenase inside live Müller cells, forming formazan dye. This dye exhibits quantifiable absorbance at a wavelength of 450 nm, serving as a proxy for the number of metabolically active cells. An immunosorbent assay reader (VERSAmax, Molecular Devices, Sunnyvale, CA, USA) was employed to measure the absorbance of the cultured cells against a background control. Six replicates were performed in each group.

### 4.6. Target Gene Prediction and Gene Ontology Enrichment Analysis

To identify potential target genes of miR-152-3p and miR-196-5p, analyses were conducted using TargetScan and miRDB [56,57]. The predicted targets were determined by the intersection of results from both algorithms and were subsequently subjected to further analysis using R and the *clusterProfiler* package [58,59]. Gene function and pathway information were assessed using the KEGG and Reactome [60,61,62]. The significance criterion for target gene identification was set at *p* < 0.05.

### 4.7. Statistical Analyses

The data obtained from the cell proliferation and migration assays were analyzed using Excel (version 16.7; Microsoft, Redmond, WA, USA). Continuous variables were compared using an independent *t* test, and a *p* value of <0.05 was considered statistically significant. 

## Figures and Tables

**Table 1:** Sequence of the synthetic microRNA mimics.

MicroRNA	Sequence
rno-miR-196a-5p	UAGGUAGUUUCAUGUUGUUGGG
rno-miR-152-3p	UCAGUGCAUGACAGAACUUGG
rno-miR-132-3p	UAACAGUCUACAGCCAUGGUCG
rno-miR-146b-5p	UGAGAACUGAAUUCCAUAGGCUGU

## Data Availability

The data presented in this study are available on request from the corresponding authors.
